# Supplementary material for: HPTLC-Based Chemical Profiling: An Approach to Monitor Plant Metabolic Expansion Caused by Fungal Endophytes
Source: Metabolites. 2021 Mar 17;11(3):174. doi: 10.3390/metabo11030174 (PMC8002819; doi:10.3390/metabo11030174)
Supplement: Supplementary file 1 [file metabolites-11-00174-s001.pdf]

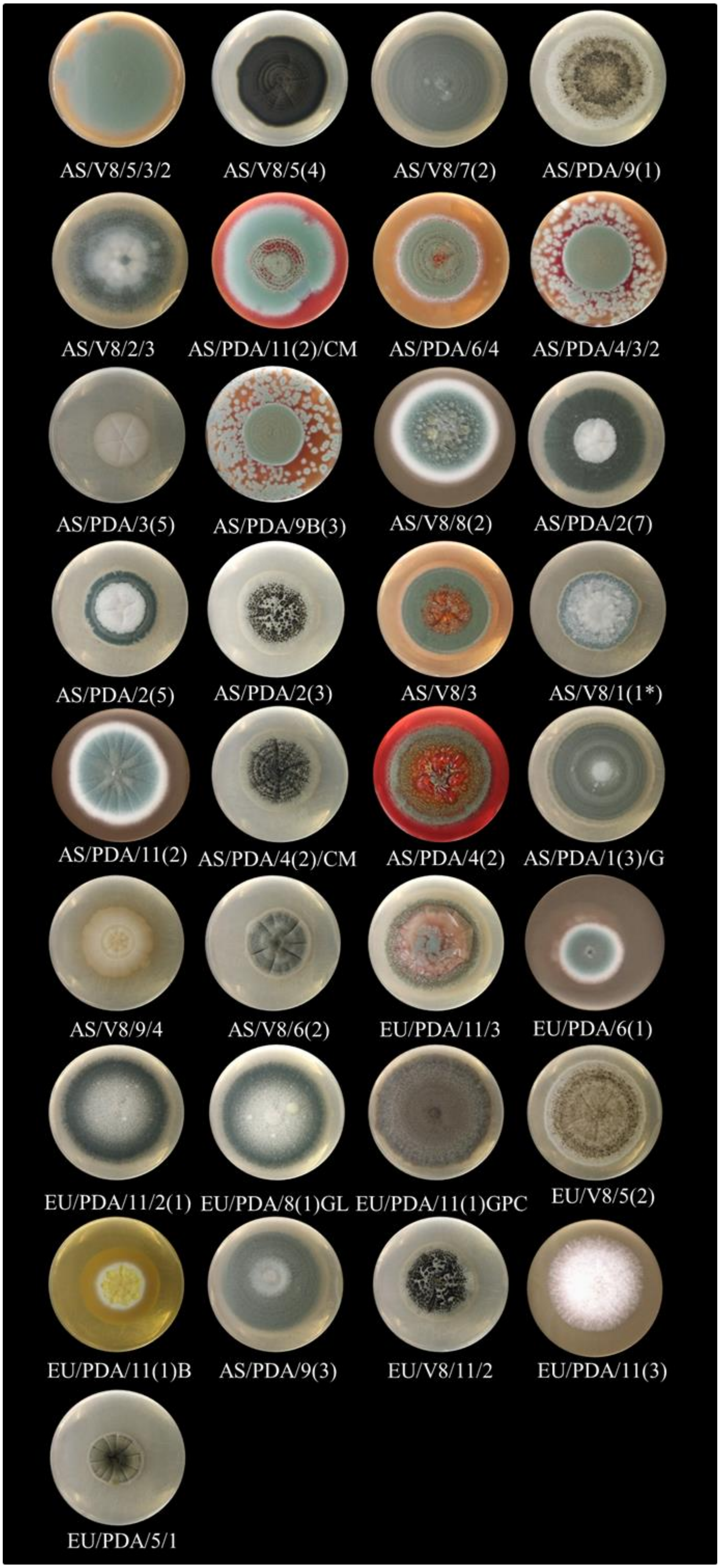

Figure S1. Fungal endophytes colonies isolated from *Alstonia scholaris* (Apocynaceae) and *Euphorbia myrsinites* (Euphorbiaceae). AS in the name of the strains means that the strain was Isolated from *S. cholaris*, and EU means isolated from *E. myrsinites*.

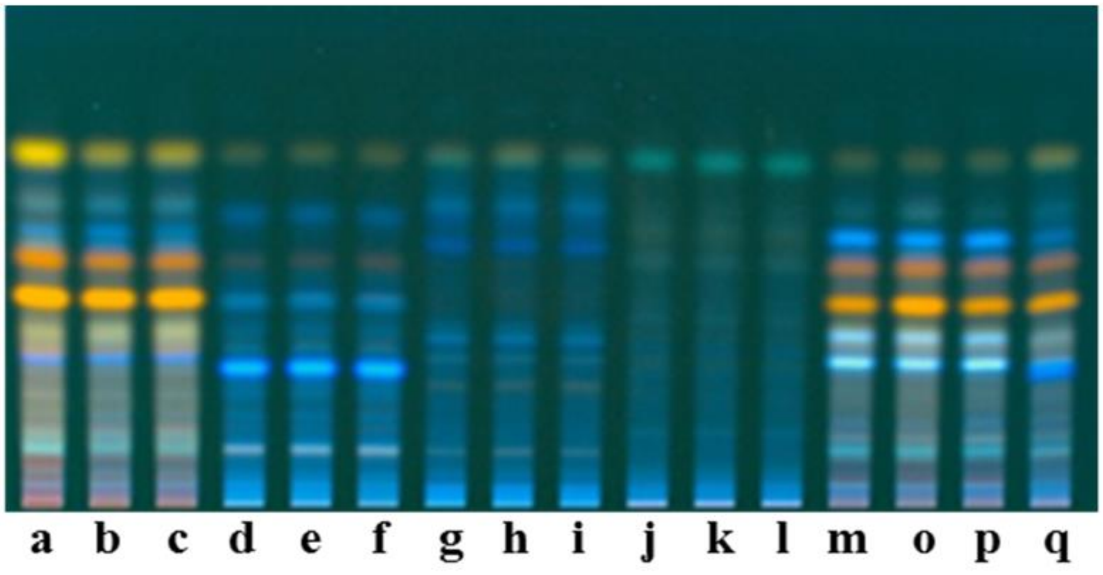

Figure S2. High performance thin layer chromatogram of five fungal endophytic strains. Each three lanes from left to right represent three biological replicates of one endophytic strain cultured on PDA. Lane "q" is a quality control sample consisting of a mix of all the extracts, which was used for further multivariate data analysis.

Table S1. Accession numbers and supplementary isolated strain information.

Accession number	Primer	Sense	Identity	Isolation source	Organ	Gene sequence
MW752807	V9g	Forward	*P. crustosum*[a]	*A. scholaris*[c]	L[e]	1 aaataaggag aaaaatattt ttggggtttg ggtttaaatt tttccccctc ctttacacac<br>61 cgcccgtcgc tactaccgat tgaatggctc agtgaggcct tgggattggc ttaggagggt<br>121 tggcaacgac cccccagagc cgaaaacttg gtcaaactcg gtcatttaga ggaagtaaaa<br>181 gtcgtaacaa ggtttccgta ggtgaacctg cggaaggatc attaccgagt gagggccctc<br>241 tgggtccaac ctccccaccc gtgtttattt taccttgttg cttcggcggg cccgccttaa<br>301 ctggccgccg gggggcttac gcccccgggc ccgcgcccgc cgaagacacc ctcgaactct<br>361 gtctgaagat tgaagtctga gtgaaaatat aaattattta aaactttcaa caacggatct<br>421 cttggttccg gcatcgatga agaacgcagc gaaatgcgat acgtaatgtg aattgcaaat<br>481 tcagtgaatc atcgagtctt tgaacgcaca ttgcgccccc tggtattccg gggggcatgc<br>541 ctgtccgagc gtcattgctg ccctcaagcc cggcttgtgt gttgggcccc gtcccccgat<br>601 ctccgggggga cgggcccgaa aggcagcggc ggcaccgcgt ccggtcctcg agcgtatggg<br>661 gctttgtcac ccgctctgta ggcccggccg gcgcttgccg atcaacccaa atttttatcc<br>721 aggttgacct cgg
MW752808	ITS4	Reverse	*P. crustosum*	*A. scholaris*	L	1 cgtaccgatt gatggctcag tgaggccttg ggattggctt tcggagggtt ggcaacgacc<br>61 ccccagtagc cgaaaacttt ggtcaaactc ggtcatttag aggaagtaaa agtcgtaaca<br>121 aggtttccgt aggtgaacct gcggaaggat cattaccgag tgagggccct ctgggtccaa<br>181 cctcccaccc gtgtttattt taccttgttg cttcggcggg cccgccttaa ctggccgccg<br>241 gggggcttac gcccccgggc ccgcgcccgc cgaagacacc ctcgaactct gtctgaagat<br>301 tgaagtctga gtgaaaatat aaattattta aaactttcaa caacggatct cttggttccg<br>361 gcatcgatga agaacgcagc gaaatgcgat acgtaatgtg aattgcaaat tcagtgaatc<br>421 atcgagtctt tgaacgcaca ttgcgccccc tggtattccg gggggcatgc ctgtccgagc<br>481 gtcattgctg ccctcaagcc cggctggtgt gttggggccc cgtcccccga tctccggggg<br>541 acgggcccga aaggcagcgg cggcaccgcg tccggtcctc gagcgtatgg ggctttgtca<br>601 cccgctctgt aggcccggcc ggcgcttgcc gatcaaccca aatttttatc caggttgacc<br>661 tcggatcagg tagggatacc cgctgaactt aagctaccta aaaaaaagaa aagaaagata<br>721 aaaaaagata aaaagggggt t
Processing	V9g	Forward	*Penicillium spp.*	*A. scholaris*	L	Processing
Processing	ITS4	Reverse	*Penicillium spp.*	*A. scholaris*	L	Processing
Processing	V9g	Forward	*Penicillium spp.*	*A. scholaris*	L	Processing
Processing	ITS4	Reverse	*Penicillium spp.*	*A. scholaris*	L	Processing
Processing	V9g	Forward	*C. acutatum*[b]	*E. myrsinites*[d]	S[f]	Processing
Processing	ITS4	Reverse	*C. acutatum*	*E. myrsinites*	S	Processing

[a] *Penicillium crustosum;* [b] *Colletotrichum acutatum;* [c] *Alstonia scholaris;* [d] *Euphorbia myrsinites;* [e] Leaf; [f] Stem.
